# Influence of therapeutic plasma exchange treatment on short-term mortality of critically ill adult patients with sepsis-induced organ dysfunction: a systematic review and meta-analysis

**DOI:** 10.1186/s13054-023-04795-x

**Published:** 2024-01-04

**Authors:** Vladimir Kuklin, Michael Sovershaev, Johan Bjerner, Philip Keith, L. Keith Scott, Owen Matthew Truscott Thomas, Wladimir Szpirt, Gail Rock, Bernd Stegmayr

**Affiliations:** 1 Department of Anaesthesiology and Intensive Care Medicine, Ahus University Hospital, Sykehusveien, 25, 1478 Lorenskog, Norway; 2 Fürst Medical Laboratory, Oslo, Norway; 3 Critical Care Medicine, Lexington Medical Center, West Columbia, SC USA; 4 Division of Trauma and Surgical Critical Care, Louisiana State University Health Sciences Center, Shreveport, USA; 5 Department of Health Services Research, Ahus University Hospital, Lorenskog, Norway; 6 Department of Nephrology, Rigshospitalet, University of Copenhagen, Copenhagen, Denmark; 7 University of Ottawa, Ottawa, ON Canada; 8 Department of Public Health and Clinical Medicine, Umea University, Umea, Sweden

## Abstract

**Introduction:**

The impact of therapeutic plasma exchange (TPE) on short-term mortality in adult patients with sepsis-induced organ dysfunction remains uncertain. The objective of the study is to assess the effect of adjunct TPE in this setting through a comprehensive literature review.

**Methods:**

The National Library of Medicine’s Medline, Ovid (Embase), the Cochrane Library database and clinicaltrial.gov from January 01, 1966, until October 01, 2022, were searched for terms: therapeutic plasma exchange, plasmapheresis, sepsis, and septic shock. We reviewed, selected and extracted data from relevant randomized clinical trials (RCTs) and matched cohort studies (MCSs) comparing short-term mortality in critically ill adult septic patients treated with standard therapy versus those receiving adjunct TPE. Risk of bias was assessed in the RCTs using Cochrane Collaboration tool and in MCSs using ROBINS-I tool. Summary statistics, risk ratios (RRs), and confidence intervals (CIs) were calculated using random effects model.

**Results:**

This systematic review included 937 adult critically ill septic patients from five RCTs (n = 367) and fifteen MCSs (n = 570). Of these total, 543 received treatment with TPE in addition to standard care. The meta-analysis includes all five RCTs and only six MCSs (n = 627). The adjunct TPE treatment (n = 300) showed a significant reduction in short-term mortality (RR 0.59, 95% CI 0.47–0.74, I2 3%) compared to standard therapy alone (n = 327). The systematic review of all 20 trials revealed that adding TPE to the standard therapy of critically ill septic patients resulted in faster clinical and/or laboratory recovery.

**Conclusions:**

Our comprehensive and up-to-date review demonstrates that adjunct TPE may provide potential survival benefits when compared to standard care for critically ill adult patients with sepsis-induced organ dysfunction. While results of this meta-analysis are encouraging, large well-designed randomized trials are required to identify the optimal patient population and TPE procedure characteristics prior to widespread adoption into practice.

**Supplementary Information:**

The online version contains supplementary material available at 10.1186/s13054-023-04795-x.

## Introduction

Sepsis is defined by the Third International Consensus as “life-threatening organ dysfunction caused by a dysregulated host response to infection” [1], and is comprised of a complex, intertwined interaction of inflammation, endothelial dysfunction, capillary leak, and a spectrum of pathologic coagulation [2]. Various treatments [3] have been previously investigated, targeting specific components of this pathological host response, but apart from rapid administration of antibiotics [4], results have been inconsistent and largely disappointing. TPE has long been hypothesized as a possible treatment [5] through simultaneous actions on multiple aspects of the pathway. Over the years, multiple case reports and case series [6–34] have produced encouraging results, demonstrating reduced short-term mortality and improved clinical outcomes in patients with sepsis-induced organ dysfunction receiving adjunct TPE. However, prospective, randomized data are scarce and inconclusive [35, 36]. Previous attempts to clarify have resulted in five meta-analyses [37–41] with the authors of these analyses concluding there is insufficient evidence to recommend TPE as routine therapy for patients with sepsis-induced organ dysfunction. Currently, the American Society for Apheresis (ASFA) 2023 guidelines [42] provide a category III, 2A recommendation for the use of TPE for patients with sepsis-induced organ dysfunction, allowing for individualized use on a case-by-case basis.

Our study aims to comprehensively review and analyze the currently available literature to re-evaluate the clinical impact of adjunct TPE on short-term mortality in critically ill adult septic patients with multiple organ dysfunction.

## Materials and methods

### Data source and search strategy

This systematic review and meta-analysis were performed by two investigators according to PRISMA (Preferred Reporting Items Systematic Reviews and Meta-Analysis) guidelines [43] and a pre-published protocol (PROSPERO database, CRD 42022377753). The National Library of Medicine’s Medline, Ovid (Embase), Cochrane library, and clinicaltrial.gov were searched for randomized, observational, and retrospective clinical studies of plasma exchange for treatment of septic patients with the following search terms: “plasma exchange, plasmapheresis, sepsis, and septic shock”. In addition, we hand searched references from retrieved articles to identify other eligible clinical studies. The search period took place from 01.01.1966 to 01.11.2022 and the language of the articles was limited to English. Initially, we planned to include only TPE by centrifuge technique. However, we deviated from the registered protocol (PROSPERO database, CRD 42022377753) due to the significant number of studies that performed TPE by filtration technique, which could have a significant impact on outcomes.

The assessed primary outcome was short-term mortality (14–35 days depending on individual study endpoints). Secondary outcomes included clinical (hemodynamics, noradrenaline dosing), laboratory, and severity of illness scores (SOFA, APACHE II, APACHE III). Post hoc subgroup analyses were performed based on TPE procedure type (membrane, centrifugal) and type of infection (non-COVID-19 versus COVID-19).

### Selection of studies

PICO inclusion criteria (Patient/Population, Intervention, Comparison, Outcome) were used for inclusion in the meta-analysis: 1. Population: critically ill adult patients with sepsis-induced multi-organ dysfunction, 2. Intervention: therapeutic plasma exchange 3. Comparison intervention: standard therapy 4. Outcomes: clinical, laboratory markers, and short-term mortality 5. Study design: randomized, controlled trials, observational and retrospective studies.

All references were independently screened at the level of abstracts by two investigators (VK, MS) and then, if fulfilling inclusion criteria, the full-text articles were obtained and reviewed.

### Data extraction and management

The first author extracted relevant information (authors, name of the article and journal, year of publication, patient demographics, illness severity scores, TPE treatment, short-term mortality, hemodynamic status/the dose of noradrenaline before and after TPE, laboratory values) from the selected articles. These data were checked independently by the second author. Discrepancies between the two investigators was resolved through consensus in discussion with the third author.

### Quality assessment

The Cochrane Collaboration tool was used to assess the risk of bias (ROB2) in the RCTs [44], and the ROBINS-I tool was used to assess the risk of bias in MCSs [45].

The Cochrane tool assesses generation of the allocation sequence, concealment of the allocation sequence, blinding (participants/personnel and outcome assessors), incomplete outcome data, selective outcome reporting, and other biases [44]. All included RCTs were evaluated by two independent reviewers for the potential risk of bias by applying a rating of “Low”, “High” or “Unclear”.

The ROBINS-I tool assesses bias due to confounding, bias in selection of participants into the study, bias in classification of interventions, bias due to deviations from intended interventions, bias due to missing data, bias in measurement of outcomes, and bias in selection of the reported result. The categories for risk of bias judgements by the ROBINS-I tool were assessed by two independent reviewers and rated a “Low risk,” or “Moderate risk,” or “Serious risk,” or “Critical risk,” or “No information.”

Two authors (VK, MS) independently reviewed the presence of authors’ possible conflict of interest and the funding source for each study, then rated each trial as of “Low,” “High,” or “Unclear” risk regarding those specific points.

### Statistical analysis

The meta-analysis was performed using the R statistical program [46], using the “meta” [47] and “robvis” [48] packages. The meta-analysis only included MCSs that compared patients receiving adjunct TPE with controls. Additionally, multiple post hoc analyses were performed for subgroups of patients treated with centrifuge versus membrane filtration techniques of TPE, as well as subgroups of COVID-19 versus non-COVID-19 patients treated by TPE. Individual trials and summary results were reported as a relative risk (RR) with 95% confidential intervals (CI) of reported mortality in patients assigned to TPE versus controls. Random effects model were reported for all analyses. A RR of less than 1 suggests a lower rate of death among patients. Statistical heterogeneity among RCTs and MCSs included in the meta-analysis was assessed and quantified using the Cochran Q test and Higgins I2 metric correspondingly. P < 0.05 was considered statistically significant. A funnel plot and Peters’ linear regression test of asymmetry were used to evaluate the risk of publication bias.

## Results

### Study selection

**Fig. 1:**
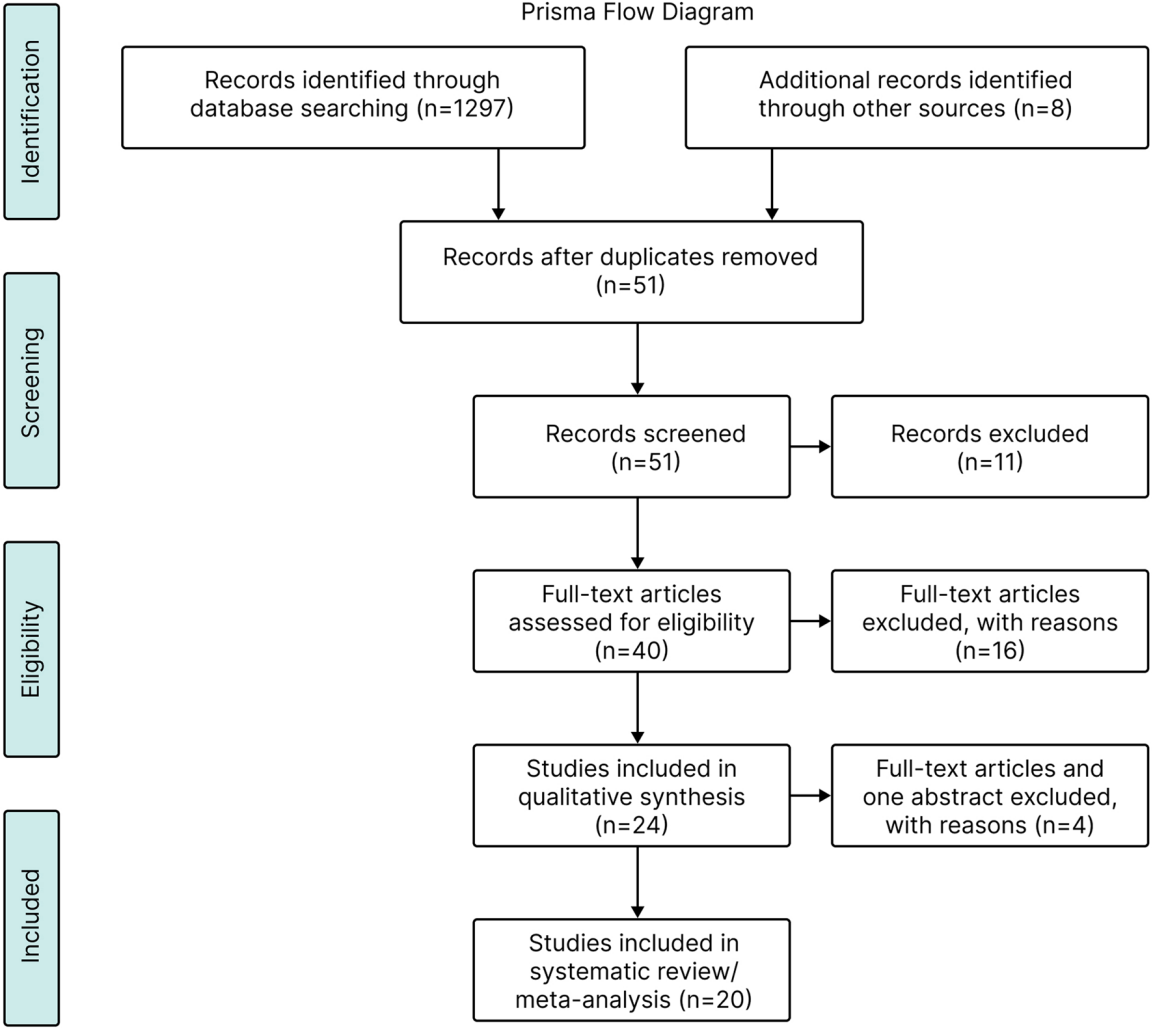
Flow diagram for the selection of clinical studies. PRISMA, Preferred reporting items systematic reviews and meta-analysis

We identified 1,305 publications from electronic and hand-searches (Fig. 1). After discarding duplicates and reviewing titles and abstracts, 1254 were excluded, leaving 51 records for analysis. Of these, 27 records were excluded for failing to meet the inclusion criteria. Of the remaining 24 records, three were excluded from analysis as they included pediatric patients, and one study was excluded because it was an abstract, leaving a total of 20 studies which were included in the analyses (5 RCTs and 15 MCSs) (Fig. 1).

The systematic review included all five randomized controlled trials (RCTs) [35, 36, 49–51] and 15 matched cohort studies (MCSs) [17, 32, 52–64], analyzing a total of 937 critically ill adult patients with sepsis and multiple organ dysfunction. Among these, 543 received adjunct therapeutic plasma exchange (TPE) in addition to standard sepsis management, while 394 patients received standard therapy alone.

The meta-analysis included only those trials comparing patients receiving adjunct TPE with controls, and included all five RCTs [35, 36, 49–51] and six MCSs [17, 55, 59, 61–63], reviewing a total of 627 critically ill adult patients with sepsis and multiple organ dysfunction. Among these, 300 received adjunct TPE in addition to standard sepsis management, while 327 patients received standard therapy alone.

### Risk of publication bias assessment

Four of the five RCTs were rated as good quality with low risk of bias, while the fifth had a high risk of bias due to selection of reported results (Table 1, Additional file 1: Fig. S1A, B). Four of the six MCS studies were rated as good quality with low risk of bias. One MCS had serious risk of bias due to confounding and a moderate risk for bias due to selection of participants. The sixth MCS had moderate risk of bias due to confounding (Additional file 1: Fig. S2A, B). Due to the nature of a TPE procedure and the severity of the general condition of septic patients, blinding of these procedures/patients from medical personnel seemed difficult and illogical, therefore we did not judge it as a crucial factor for RCTs or MCSs.

**Table 1:** Risk of bias among randomized clinical trial

Study	Risk of bias^a^						
	In randomization process	Due to effect of assignment and intervention	Due to effect of adhering to intervention	Due to missing outcome data	In measurement of the outcome	Due to selection of reported result	Due to overall risk
Reeves [35]/1999	Low	Low	Low	Low	Low	Low	Unclear
Busund [36]/2002	Low	Low	Low	Low	Low	High	High
Faquhia [49]/2021	Low	Low	Low	Low	Low	Low	Low
Weng [50]/2021	Low	Low	Low	Low	Low	Low	Low
Stahl [51]/2022	Low	Low	Low	Low	Low	Low	Low

^a^Risk of Bias was assessed using the Cochrane Risk of Bias 2 tool

We further evaluated the risk of publication bias with a funnel plot (Additional file 1: Fig. S3) and observed that the larger studies are evenly distributed around the random effects model estimate, while the smaller studies appear to be biased toward the larger effect sizes. The Peters’ regression test for funnel plot asymmetry [65] did not return a significant result (t = − 0.69, p = 0.507), indicating a low risk of publication bias (Table 2).

**Table 2:** Risk of bias among matched cohort studies

Study	Risk of bias^a^						
	Due to Confounding	In Selection	In measurement classification of interventions	Due to deviations from intended interventions	Due to missing data	In measurement of outcomes	In selection of the reported results
Gårdlund [23]/1993	Low	Low	Low	Low	Low	Low	Low
Stegmayr [52]/1995	Moderate	Moderate	Low	Low	Low	Low	Low
Stegmayr [53]/1996	Low	Low	Low	Low	Low	Low	Low
Hjorth [54]/2000	Low	Low	Low	Low	Low	Low	Low
Schmidt [55]/2000	Serious	Moderate	Low	Low	Low	Low	Low
Ataman [56]/2002	Serious	Serious	Moderate	Low	Low	Low	Low
Stegmayr [57]/2003	Moderate	Low	Low	Low	Low	Low	Low
Hadem [32]/2014	Low	Low	Low	Low	Low	Low	Low
Knaup [58]/2018	Low	Low	Low	Low	Low	Low	Low
Keith [59]/2020	Low	Low	Low	Low	Low	Low	Low
Ahmed [60]/2020	Low	Low	Low	Low	Low	Low	Low
Gucyetmez [61]/2020	Moderate	Low	Low	Low	Low	Low	Low
Khamis [62]/2020	Low	Low	Low	Low	Low	Low	Low
Kamran [63]/2021	Low	Low	Low	Low	Low	Low	Low
Jaiswal [64]/2021	Low	Low	Low	Low	Low	Low	Low

^a^Risk of bias was assessed using the Risk of Bias in Non-Randomized Studies of interventions (ROBINS-I) for interventional studies. “Low risk,” “Moderate risk,” “Serious risk,” “Critical risk,” and “No information”

### Characteristics and primary outcome of clinical studies

The median trial size was 43 patients, ranging from 7 to 106 participants. With the exception of one RCT, [36] (difference in mean age), there were no significant differences in baseline characteristics between controls and patients who underwent adjunct TPE in the RCTs. Inclusion criteria, TPE technique, choice and volume of replacement fluid, and number of TPE treatments varied among the trials (Table 3).

**Table 3:** Trials characteristics

The first author of study/year of publication	Type of study	Sample size	Major inclusion criteria	Volume and type of TPE	Number of TPE session	TPE group 14–35 day Mortality, n (%)	Control group 14–35 day Mortality, n (%)	P-value
Reeves [35]/1999	RCT	22	Severe sepsis	100–150 ml/kg F	2–3	3/9 (33.3%) at 14 day	6/13 (46.1%)	
Busund [36]/2002	RCT	106	Severe sepsis, septic shock	30–40 ml/kg C	1–2	18/54 (33.3%) at 28 day	28/52 (53.8%)	0.05
Faquhia [49]/2021	RCT	87	Critically ill COVID-19, MOF, N/A infusion dose > 0.4	30–40 ml/kg C	1–5	9/43 (20.9%) at 35 day	15/44 (34.1%)	0.62
Weng [50]/2021	RCT	112	Severe sepsis, septic shock, DIC	30 ml/kg F	1–3	6/40 (15%) at 28 day	14/36 (38.8%)	< 0.05
Stahl [51]/2022	RCT	40	Septic shock with onset < 24 h, N/A infusion dose > 0.4	45 ml/kg C	1	8/20 (40%) at 28 day	10/20 (50%)	0.43
Gårdlund [17]/993	PS	28	Septic shock	30 ml/kg F	1	1/7 (14%) at 28 day	Historical control 8/21 (38%)	Nr
Stegmayr [52]/1995	PS	27	Severe sepsis, septic shock, MOF	30–35 ml/kg C	1–10	5/27 (19%) at discharge from hospital	Nr	Nr
Stegmayr [53]/1996	PS	25	Severe sepsis, septic shock	30–35 ml/kgC	1–10	5/25 (20%) at discharge from hospital	Predicted mortality 80%	< 0.001
Hjorth [54]/2000	RS	17	Septic shock	30–40 ml/kg C	1–2	3/17 (18%) at 28 day	Calculated mortality based on initial APACHE II (62%)	0.01
Schmidt [55]/2000	PS	43	Severe sepsis	nr F	1	8/19 (42.1%) at 28 day	11/24 (45.8%)	Ns
Ataman[56]/2002	RS	7	N/A-refractory septic shock	30–40 ml/kg F	1–2	6/7 (85.7%) at 8 day	Nr	Nr
Stegmayr [57]/2003	RS	76	Severe sepsis, septic shock	30–35 ml/kg C	1–14	16/76 (18.4%) at discharge from hospital	Calculated mortality based on initial APACHE II (82%)	0.0001
Hadem [32]/2014	RS	23	Severe sepsis, septic shock	45 ml/kg F	1–3	9/23 (39%) not reported	Nr	Nr
Knaup [58]/2018	PS	20	Septic shock with onset < 24 h, N/A infusion dose > 0.4	30–35 ml/kg F	1	13/20 (65%) at 28 day	Nr	Nr
Keith [59]/2020	RS	80	Known source of infection, MOF (> 2), infusion of two or more pressors	30–35 ml/kg C	1–2	16/40 (40%) at 28 day	26/40 (65%)	0.043
Ahmed [60]/2020	PS	16	Septic shock, N/A infusion dose > 0.4	30 ml/kg F	1–3	10/16 (62.5%) not reported	Calculated mortality based on initial APACHE II (86.7%)	Nr
Gucyetmez [61]/2020	PS	73	Critically ill COVID-19, MOF	nr C	3	1/12 (8.3%) at 35 day	7/12 (58.3%)	0.009
Khamis [62]/2020	PS	31	Critically ill COVID-19, MOF, N/A infusion dose > 0.4	30–40 ml/kg C	5	0/11 at 28 day	7/20	0.033
Kamran [63]/2021	RS	90	Critically ill COVID-19, MOF	45 ml/kg C	1–5	4/45 (8.8%) at 28 day	28/45 (61.5%)	< 0.001
Jaiswal [64]/2021	PS	14	Critically ill COVID-19, MOF	30–40 ml/kg C	1	4/14 (28.6%) at 28 day	Nr	Nr

**Fig. 2:**
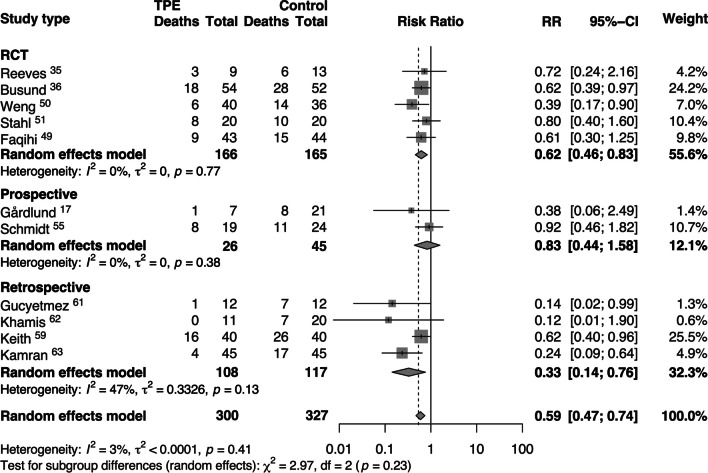
Risk ratios (RRs) of short-term mortality associated with therapeutic plasma exchange (TPE) treatment versus standard treatment of septic patients. Pooled risk ratios are from random effects model. Boxes and horizontal lines represent point estimates, varying in size according to the weight in the analysis, and 95% confidence intervals (CI). X^2^ = Chi-squared; df = degrees of freedom; *I*^*2*^ = *I*-squared; *t*^2^ = Tau-squared; *Z* = *Z* score; *p* = probability value

The five RCTs included 331 septic patients [35, 36, 49–51] with 166 receiving adjunct TPE (Table 3). Among these trials, patients treated with adjunct TPE had a lower mortality rate (RR: 0.62 [95% CI: 0.46, 0.83]) compared to those who received standard therapy alone (Fig. 2). In three RCTs [36, 49, 51], TPE was performed by centrifuge technique and the volume of plasma removed and replaced was between 30 and 45 ml/kg (Table 3). Membrane filtration was used in two RCTs [35, 50], and 30–150 ml/kg of plasma was removed (Table 3).

Five hundred and seventy patients were included in the 15 MCSs [17, 28, 52–64] with 377 of these patients receiving adjunct TPE (Table 3). Compared to those receiving standard therapy alone, patients receiving adjunct TPE had a non-significant decreased risk of short-term mortality in the subgroup of prospective studies (RR: 0.83 [95% CI: 0.44, 1.58]), while there was a significant reduction in the risk of short-term mortality in the subgroup of retrospective studies (RR: 0.33 [95% CI: 0.14, 0.76], Fig. 2). TPE by centrifugation was used in nine studies [52–54, 57, 59, 61–64] with removal and replacement of 30–45 ml/kg of plasma. The remaining six MCSs [17, 28, 55, 56, 58, 60] utilized the filtration technique, with an average of 30–45 ml/kg of plasma removed (Table 3).

### Meta-analyses of pooled short-term mortality and subgroup analyses

We pooled data on short-term mortality from the 5 RCTs and 6 MCSs (n = 627) comparing those receiving standard therapy (n = 327) to those treated with adjunct TPE in addition to standard therapy (n = 300). The random effects model showed a significant reduction in the risk ratio of death (RR 0.59, [95% CI 0.47, 0.74]), with low overall heterogeneity (I^2^ = 3%, τ^2^ < 0.0001; Fig. 2).

**Fig. 3:**
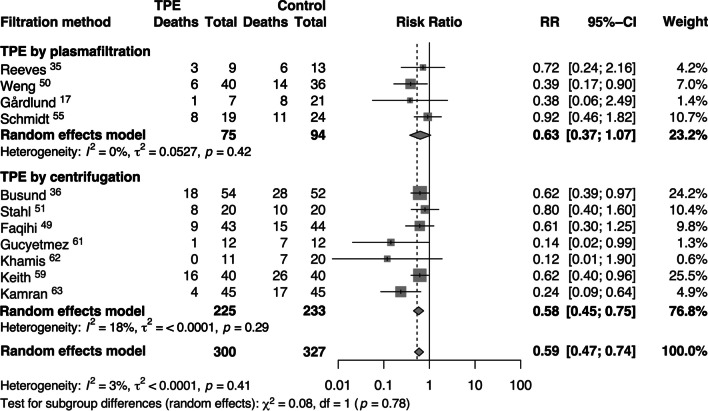
Risk ratios (RRs) of short-term mortality associated with membrane filtration and centrifuge techniques of therapeutic plasma exchange (TPE) treatment in septic patients compared to standard treatment. Pooled risk ratios are from random effects model. Boxes and horizontal lines represent point estimates, varying in size according to the weight in the analysis, and 95% confidence intervals (CI). X^2^ = Chi-squared; df = degrees of freedom; *I*^*2*^ = *I*-squared; *t*^2^ = Tau-squared; *Z* = *Z* score; *p* = probability value

We performed subgroup analyses by TPE method used. Filtration technique demonstrated a non-significant mortality reduction through random effects analysis (RR = 0.63, [95% CI 0.37, 1.07]) and very low heterogeneity (I^2^ = 0%, τ^2^ = 0.0527). In comparison, centrifugation technique demonstrated a decreased risk of short-term mortality with greater effect size and significance through random effects models (RR = 0.58, [95% CI 0.45, 0.75]) with quite low heterogeneity (I^2^ = 18%, τ^2^ < 0.0001), (Fig. 3).

**Fig. 4:**
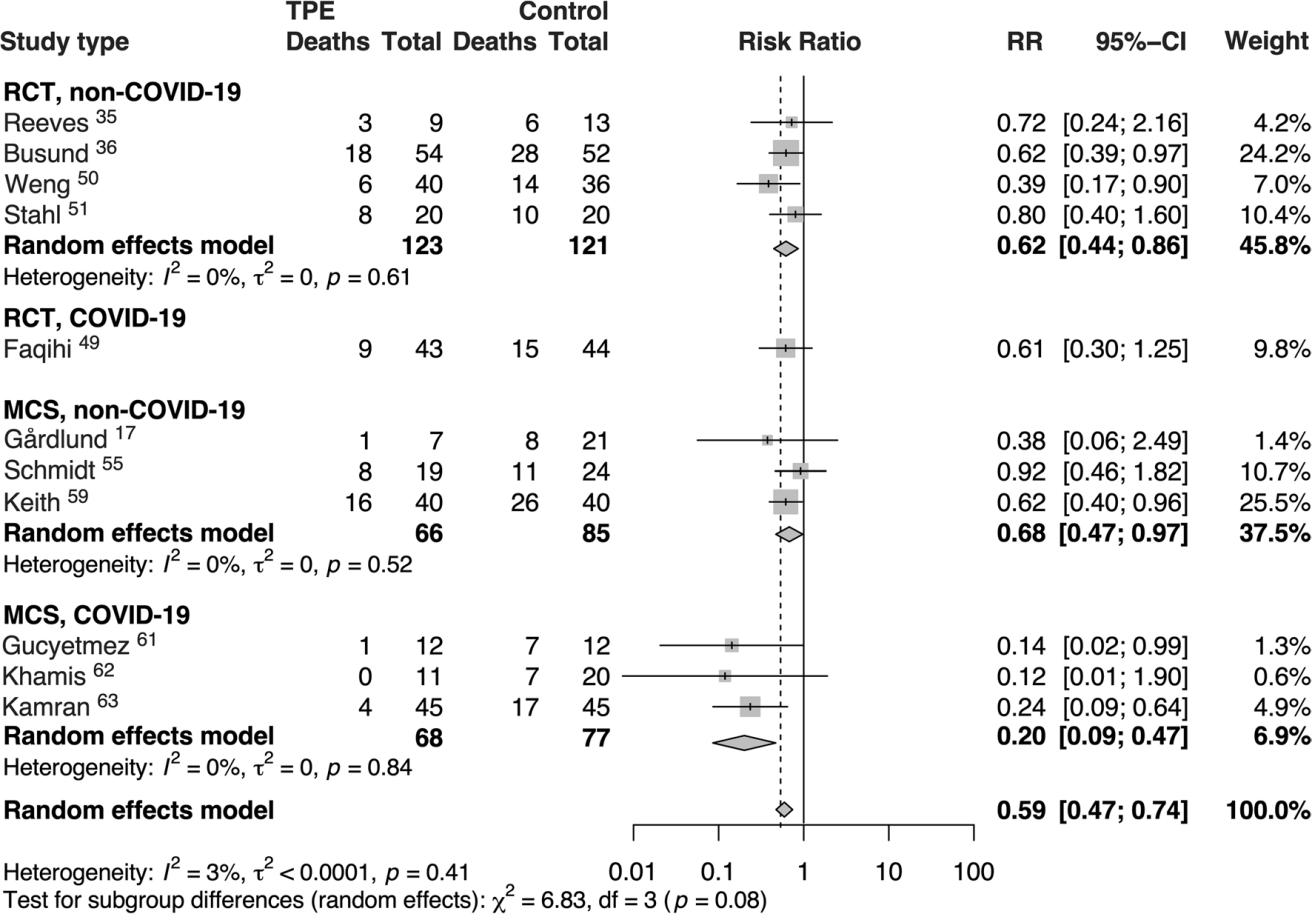
Risk ratios (RRs) of short-term mortality in septic patients with and without COVID-19 receiving therapeutic plasma exchange (TPE) or standard treatment. Pooled risk ratios are from random effects model. Boxes and horizontal lines represent point estimates, varying in size according to the weight in the analysis, and 95% confidence intervals (CI). X^2^ = Chi-squared; df = degrees of freedom; *I*^*2*^ = *I*-squared; *t*^2^ = Tau-squared; *Z* = *Z* score; *p* = probability value

We also conducted a subgroup analysis comparing patients with sepsis caused by COVID-19 and those with sepsis from non-COVID-19 etiology (Fig. 4). Patients with sepsis caused by pathogens other than COVID-19 (referred to as non-COVID-19 sepsis) showed a reduced risk of short-term mortality in both randomized controlled trials (RCTs) (RR = 0.62, [95% CI 0.44, 0.86]) and observational studies (MCSs) (RR = 0.68, [95% CI 0.47, 0.97]). The random effects model indicated low heterogeneity (I^2^ = 0%, τ^2^ = 0) (Fig. 4). On the other hand, patients with sepsis due to COVID-19 exhibited a decreased risk of short-term mortality in MCSs (RR = 0.20, [95% CI 0.09, 0.47]) with low heterogeneity (I^2^ = 0%, τ^2^ = 0) (Fig. 4).

### Secondary clinical and laboratory outcomes

Multiple studies reported potentially relevant clinical and laboratory endpoints. Three RCTs [49–51] reported a significant reduction in noradrenaline doses required to maintain goal arterial blood pressure (ABP) following TPE. Two MCSs [58, 60] showed that TPE significantly improved the mean arterial pressure and stroke volume variance, enabling reduced noradrenaline doses. One MCS [56] found no change in hemodynamic status after TPE.

A significant reduction in Acute Physiology and Chronic Health Evaluation (APACHE) II [50], III [36] and Sequential Organ Failure Assessment (SOFA) [49, 50] scores following TPE were observed in four RCTs [35, 36, 49, 50], while one MCS [59] demonstrated a significant reduction in the SOFA score following TPE. One MCS [55] showed no change in the APACHE II score after TPE.

Three RCTs [49–51] demonstrated a significant decline in the plasma concentration of inflammatory cytokines, immune antibodies, serum lactate, lactate dehydrogenase (LDH), ferritin, D-dimer, and injurious mediators such as procalcitonin (PCT), von Willebrand factor antigen (vWF:Ag), angiopoietin-2 (Angpt-2) and a soluble receptor of tyrosine kinase with immunoglobulin-like and EGF-like domains (sTie-2) following TPE. In these trials, TPE also resulted in a significant increase in the number of lymphocytes, platelets, and repletion of protective factors such as antithrombin-III (AT III), protein C and a disintegrin and metalloprotease with thrombospondin type 1 motif 13 (ADAMTS-13) [49–51].

Two MCSs [56, 58] demonstrated a significant reduction in plasma concentration of inflammatory cytokines (interleukin (IL)-1 [58], IL-6 [58, 61], IL-8 [58], IL-10 [58]), C-reactive protein [61], procalcitonin [61], D-dimer [61], ferritin [61], and LDH [61] following TPE.

## Discussion

Our meta-analysis demonstrates a significant reduction in short-term mortality when adjunct TPE is added to the standard therapy of critically ill patients with sepsis-induced organ dysfunction. These findings, combined with those of three recently published meta-analyses [66–68] add to the current body of evidence reflected in the 2023 ASFA guidelines which allow TPE to be considered on a case to case basis for sepsis with multiple organ dysfunction (category III, 2A recommendation) [41]. In addition, a large propensity-score matched analysis [69] demonstrating reduced 28-day and 1-year mortality associated with TPE in septic patients with MODS was published in November 2023, but was not included in the current analysis so as to avoid deviations from the predefined protocol and to avoid bias.

While these results are encouraging, it is important to acknowledge and address several limitations. As with any trial that includes retrospective observational reports, there is inherent bias that cannot be completely eliminated. It is again worth noting that in each trial, including the RCTs, a “sham” intervention was not performed because TPE requires an intervention and treatment that is difficult to “blind” from clinical providers. This lack of blinding could lead to bias among the treatment teams, which could affect management and would be difficult to eliminate due to the logistics of the intervention. Additionally, authors with negative and/or equivocal outcomes are less likely to publish their findings, so these outcomes may not be reported. In our efforts to identify published and unpublished studies, we conducted extensive searches of several databases, identifying those studies referenced in Table 3. In addition, we evaluated the risk of publication bias with a funnel plot (Additional file 1: Fig. S3), and the Peters’ regression test for funnel plot asymmetry [65] indicated a low risk of publication bias (*t* = − 0.69, p = 0.507). While these measures cannot guarantee that conflicting outcomes have not been observed clinically, our literature search and analysis were comprehensive.

By the nature of their design, meta-analyses and systematic reviews are limited by differing treatment protocols/algorithms and variable outcome measures among the included trials. While trials in critical care typically report 28–30 day mortality [2], the trials included in this analysis varied from 14 to 35 days (Table 3). Furthermore, the actual day of death is not reported in the individual trials, rather the data includes only short-term death or short-term survival as the outcome (Table 3). The authors of the current manuscript do not have access to outcomes beyond those reported in the original manuscripts, making it impossible to analyze whether the effect on mortality would differ if the same mortality endpoint were used in all trials.

Additionally, while long-term outcomes would be desirable, the design and endpoints of the included trials do not allow for the assessment of outcomes beyond 35 days.

A major limitation of our current review is the inability to assess the timing of TPE on mortality. The hallmark of sepsis management is early therapy, and it would seem intuitive that timely initiation of TPE would be paramount to response. Only one of the included trials included strict criteria in terms of timing, and none reported outcomes in relation to time. Similarly, a great deal of heterogeneity exists among the trials in terms of TPE eligibility criteria. Sepsis diagnostic criteria have evolved, and quantifying/analyzing the severity of illness among the trials is not possible with the available data. The absence of this information limits the generalizability of the results and should be a priority in future prospective trials.

The review is further limited by the variability in the number of TPE treatments performed (Table 3). Unfortunately, further analysis is not possible with the data available from each individual trial. Based on the authors’ clinical experience, we support that the number of procedures should be based on clinical response and not pre-determined. Although this cannot be confirmed or refuted by the current literature, this strategy is supported by the low incidence of severe adverse events attributed to the TPE treatment [70]. Future prospective trials should include this topic.

Guidelines and protocols also varied across the included trials in terms of technique. Both membrane and centrifuge modalities effectively remove pathological macromolecules, but some differences are worth noting. The membrane filtration technique has a lower plasma extraction ratio, requiring higher blood flow rates and a longer procedure. The centrifugation technique removes extracellular vesicles (EV) expelled as inflammatory mediators into plasma [71], while the membrane filtration technique results in partial deposition of the EV in the filter [72]. Additionally, the blood-membrane interaction itself activates cells and inflammation, which may require more procedures to achieve down-regulation of inflammation [73]. A subgroup analysis of TPE techniques suggests different efficacies of these techniques (Fig. 3). Future studies should include an emphasis on the efficacy of each technique.

The type and volume of replacement fluid also varied among the trials and may have impacted outcomes. Sepsis is characterized by decreased ADAMTS-13 activity, which results in increased thrombogenic ultra-large von Willebrand factor (ULvWF) multimers and potentially diffuse microcirculatory platelet thrombosis [2, 3]. Increased plasminogen activator inhibitor (PAI-1) activity leads to decreased fibrinolysis and disseminated fibrin-rich microcirculatory clotting [73–75]. The net result is a non-consumptive, platelet- and fibrin-rich microcirculatory thrombotic state with non-specific coagulation findings, often distinct from other thrombotic conditions.

Replacing plasma with that from healthy donors is crucial for replenishing essential protective anti-inflammatory mediators and coagulation factors, including ADAMTS-13, which may lead to improved tissue perfusion and recovery of organ dysfunction [51, 58]. Prior studies [21] have also identified circulating markers of endothelial injury that have been associated with electron microscopic changes to the endothelium [58]. Hypotension results not only from inflammatory vasodilation, but also from increased vascular permeability caused by endothelial injury [58]. Resuscitation with fresh frozen plasma (FFP) during the TPE procedure has shown restoration of endothelial integrity as assessed by improved levels of these circulating markers and an improved microscopic appearance of the endothelium [58]. Upon clinical response, retained fluid may redistribute into pulmonary edema. Fluid removal by dialysis now is necessary unless adequate diuresis exists.

Several studies [49–51, 58, 60] have demonstrated improved hemodynamics immediately following TPE, perhaps due to this effect on the endothelium, and as a result, may allow for management with a lower volume of intravenous fluids and lower doses of vasopressors—both of which are associated with improved survival and increased ventilator-free days [49]. Thus, while the current review is unable to address these differences, the authors strongly recommend using FFP as replacement fluid when performing TPE for sepsis (as reflected in the 2023 ASFA guidelines). Some data analyzed in our review is derived from studies in patients with COVID-19-induced sepsis, and Fig. 4 shows decreased short-term mortality in those receiving adjunct TPE. While effective vaccines and therapeutics have drastically reduced the number of patients developing critical illness from COVID-19, a small percentage will still develop sepsis. The focus of this review is on the treatment of sepsis with multiple organ dysfunction, not on specific inciting pathogens. Adjunct therapy, including TPE, should not be administered solely due to COVID-19 infection, but could be considered in cases of sepsis with MODS.

Despite limitations, our review found a decreased short-term mortality in critically ill patients with sepsis and MODS receiving adjunct TPE, regardless of inciting pathogen (Fig. 4). In the absence of well-designed, prospective, double-blinded RCTs, the clinical significance of these results should not be ignored. Rather, our findings, and the limitations observed, provide a solid foundation and an urgency for future studies.

## Conclusions

Despite the small size of trials and heterogeneity of critically ill patients with sepsis and MODS, our meta-analysis demonstrates that adjunct therapeutic plasma exchange (TPE) using healthy donor plasma as replacement fluid is associated with a decreased risk of short-term mortality. While the results of this meta-analysis are encouraging, large, well-designed randomized trials are required to identify the optimal patient population and characteristics of TPE procedures prior to widespread adoption into practice.

### Supplementary Information

**Additional file 1:** Supplemental figures and figure legends.
