# Supplementary material for: Influence of therapeutic plasma exchange treatment on short-term mortality of critically ill adult patients with sepsis-induced organ dysfunction: a systematic review and meta-analysis
Source: Crit Care. 2024 Jan 4;28:12. doi: 10.1186/s13054-023-04795-x (PMC10768220; doi:10.1186/s13054-023-04795-x)
Supplement: Supplementary file 1 — Additional file 1: Supplemental figures and figure legends. [file 13054_2023_4795_MOESM1_ESM.docx]

**Supplemental figures and figure legends**

**Supplemental figure 1**. Risk of bias domains (A) and summary plot (B) among randomized controlled trials based on the ROB2 assessment tool.

A


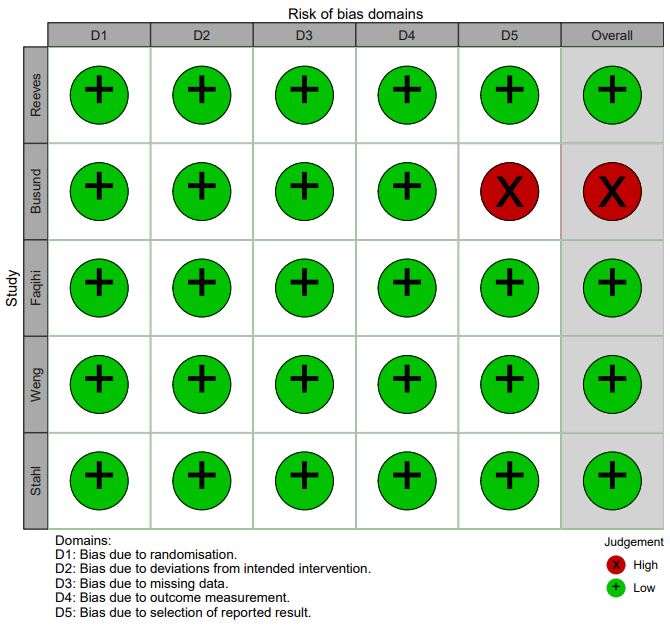


B


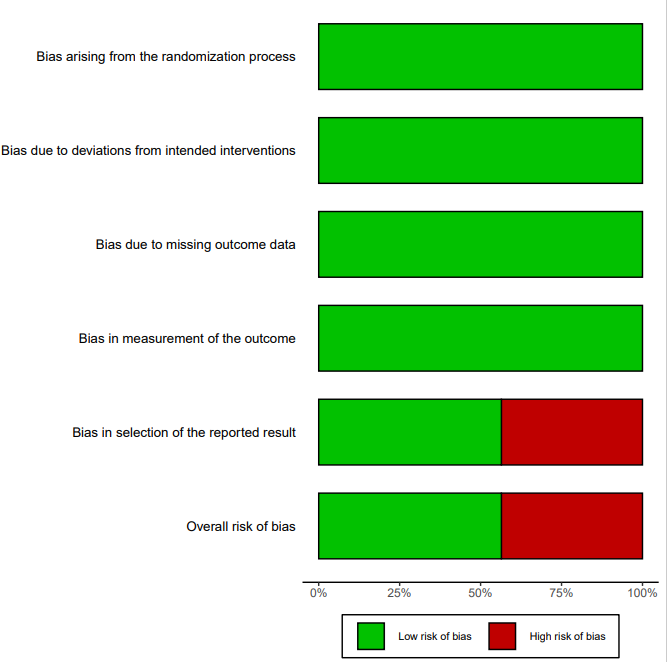


**Supplemental figure 2**. Risk of bias domains (A) and summary plot (B) of the matched cohort studies based on ROBINS-I assessment tool.

A


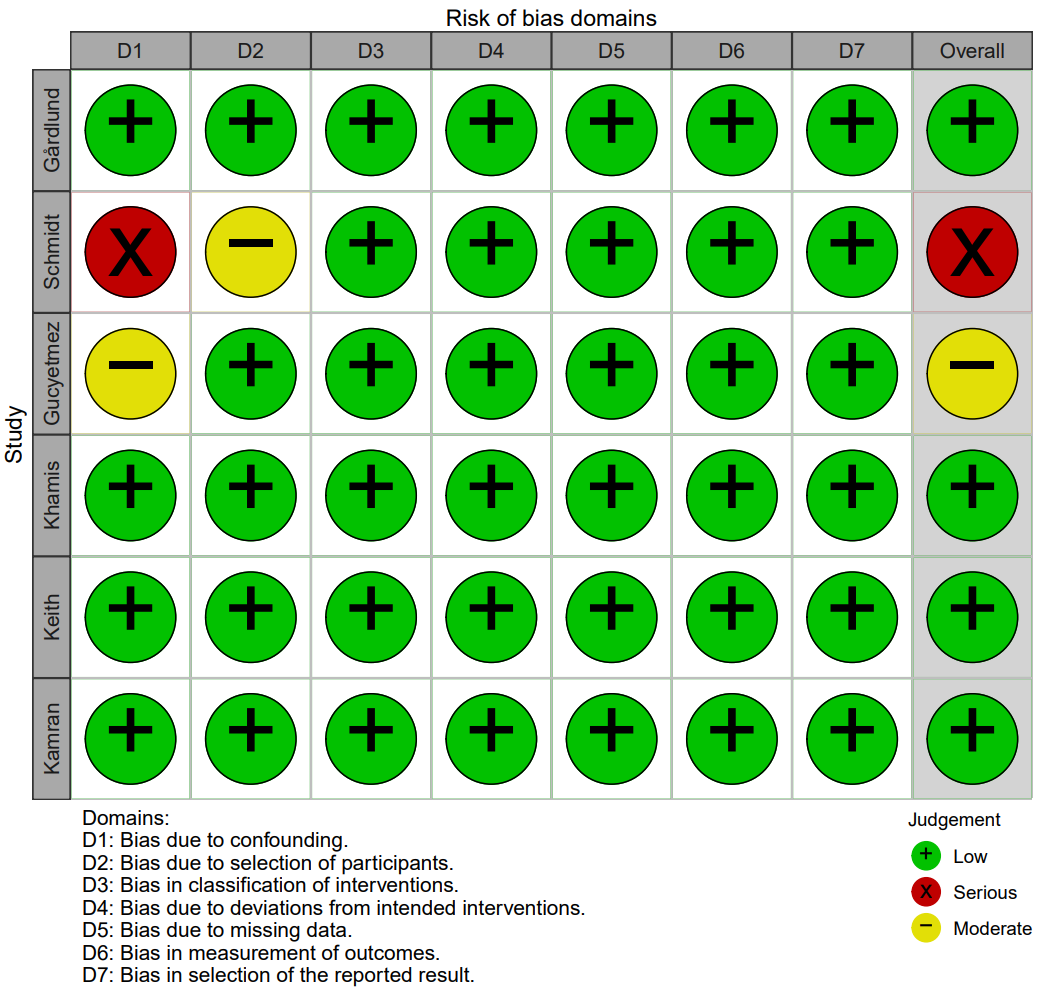


B


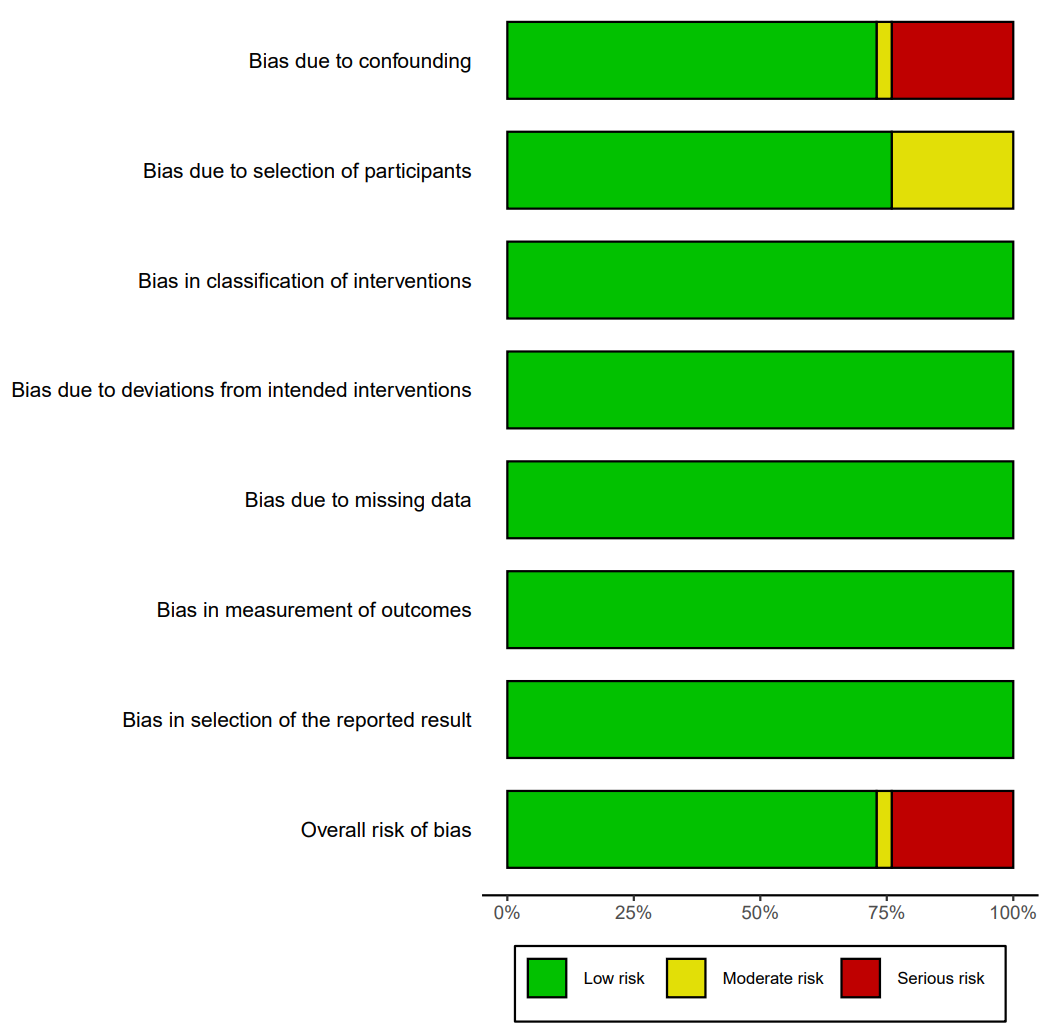


**Supplemental figure 3.** Funnel plot of Risk Ratios of short-term mortality associated with therapeutic plasma exchange (TPE) treatment versus reported Standard Errors. The dashed vertical and diagonal lines represent the common effects model estimate with projected 95% confidence interval. The dotted vertical line represents the random effects estimate. The shaded areas represent the confidence regions around the null effect (RR=1) at the 90, 95 and 99 percent confidence intervals (CI).
